# Benefits of genome-edited crops: expert opinion

**DOI:** 10.1007/s11248-019-00118-5

**Published:** 2019-03-04

**Authors:** Rim Lassoued, Diego Maximiliano Macall, Hayley Hesseln, Peter W. B. Phillips, Stuart J. Smyth

**Affiliations:** 1 Department of Agricultural and Resource Economics, University of Saskatchewan, 51 Campus Drive, Saskatoon, SK S7N 5A8 Canada; 2 The Johnson Shoyama Graduate School of Public Policy, University of Saskatchewan, 101 Diefenbaker Place, Saskatoon, SK S7N 5B8 Canada

**Keywords:** Agricultural biotechnology, Conventional crops, Genetically modified crops, Innovation, New breeding techniques, Regulation

## Abstract

Innovation in agriculture is pervasive. However, in spite of the success stories of twentieth century plant breeding, the twenty-first century has ushered in a set of challenges that solutions from the past century are unlikely to address. However, sustained research and the amalgamation of a number of disciplines has resulted in new breeding techniques (NBTs), such as genome editing, which offer the promise of new opportunities to resolve some of the issues. Here we present the results of an expert survey on the added potential benefits of genome-edited crops compared to those developed through genetic modification (GM) and conventional breeding. Overall, survey results reveal a consensus among experts on the enhanced agronomic performance and product quality of genome-edited crops over alternatives. The majority of experts indicated that the regulations for health and safety, followed by export markets, consumers, and the media play a major role in determining where and how NBTs, including genome editing, will be developed and used in agriculture. Further research is needed to gauge expert opinion after the Court of Justice of the European Union ruling establishing that site-specific mutagenic breeding technologies are to be regulated in the same fashion as GM crops, regardless of whether foreign DNA is present in the final variety.

**Electronic supplementary material:**

The online version of this article (10.1007/s11248-019-00118-5) contains supplementary material, which is available to authorized users.

## Introduction

Agriculture has benefited from technology and science through the gradual adoption of chemicals, improved crop varieties and sophisticated machines. Among the economic benefits of such innovations are increased food and feed production and cost reductions for both producers and consumers. Indeed, innovation, which is intrinsic to agriculture, is gradually increasing in sophistication, particularly innovations embedded in plants themselves. Over the last three decades, agricultural biotechnology research has extended beyond input-trait genetically-modified (GM) products and expanded into the commercialization of output-trait GM products. This development is due in part to a number of emerging new breeding techniques (NBTs), such as genome editing. However, as with most biotechnological innovations, particularly those related to food, countries assimilate or reject them based on distinct socio-economic and political realities. In the specific case of new biotechnologies, thus far, developments have been the same (Schuttelaar & Partners 2015).

Unlike complex, imprecise and lengthy conventional (CONV) breeding, genome editing led by CRISPR (Clustered Regularly Interspaced Short Palindromic Repeats) has the potential to accelerate crop improvement and food production (Yin et al. 2017). Genome editing technology allows for the targeted, and high-precision rearrangement of plant genomes (Puchta 2017). Based on preliminary applications and the biological concepts underlying genome editing, many in the scientific community are optimistic that it will contribute significantly to precision breeding, thus reducing product development costs in comparison to CONV and genetic modification (Bortesi and Fischer 2015; Georges and Ray 2017). Various techniques based on genome editing concepts offer new opportunities to develop crops with a wide spectrum of improvements at reduced costs through the clear-cut insertion of favourable traits or the knock-out or deletion of undesirable traits (Abdallah et al. 2015).

Genome-edited crops promise a host of benefits for consumers. Examples include soybeans with improved oil profiles, tomatoes with enhanced flavor qualities, non-browning apples, potatoes, and white mushrooms, and fish with enhanced muscle mass. However, diverging social perspectives within and among key consuming markets about safety, as well as potential environmental risks of genome-edited crops and foods do not bode well for consumer acceptance and subsequent regulatory approval (Ishii and Araki 2016a).

This paper reports the results of a survey that solicited opinions of experts on the potential benefits of genome-edited crops compared to their GM and CONV counterparts. Experts were asked about their opinion on several agronomic, environmental, and socio-economic benefits of genome-edited crops. The results were also tested for the effects of region of residence and type of expertise (scientific experts versus social scientists) on the perception of these benefits. The paper is structured as follows: first, we briefly review the key benefits and challenges of different breeding approaches (GM, CONV and genome editing). Next, the methodology and survey design are described, after which survey results are presented and discussed. A brief conclusion summarizes the study.

### Overview of different breeding approaches

Plant breeding began as early as 13,000 years ago when plants were first cultivated for human use (Balter 2007). It was not until 1900, with the validation of Gregor Mendel’s work on genetics, that scientific breeding methods were employed. Thanks to this work, throughout the twentieth century, development of new crop cultivars with higher yields, improved quality and better resilience was possible (Bradshaw 2017). However, for all the success stories of twentieth century plant breeding, the twenty-first century has ushered in a set of challenges that solutions from the past century are unlikely to address (Stamp and Visser 2012). Extreme climate variability, increasing water scarcity and less arable land are just some of the novel challenges plant breeders must face. It is also expected that plant breeding, at least in part, will be required to consider and respond to socio-economic factors that have never before been a concern, such as the inexorably rising demand from the increasing human population and an accompanying ensemble of factors including changing diets (influencing type, quantity and quality of food demanded), increased urbanization, and the corporate concentration of plant breeding. At the same time, biological constraints considerably limit ‘classical’ approaches to breeding, thereby giving rise to a greater need for novel breeding techniques.

#### GM crops

In 1953, the molecular structure of deoxyribonucleic acid (DNA), the chemical carrier of genetic information, was published (Watson and Crick 1953). Just over two decades later, Cohen et al. (1973) described the method with which functional foreign DNA could be inserted into another organism. This breakthrough became the foundation of genetic modification, arguably one of the most important recent developments in science, especially to modern agriculture. Thus far, genetic modification has primarily been used to introduce foreign DNA into target crops to make them insect resistant or herbicide tolerant, with these two traits often being ‘stacked’ (ISAAA 2017).

Globally, over the past two decades GM crops have provided farmers in adopting countries an array of economic, environmental, and health benefits (Smyth et al. 2015). GM crops have contributed significantly to the reduction of environmental impacts from herbicide and insecticide use. Since 1996, the use of pesticides on the GM crop area has decreased by 671.4 million kg of active ingredient relative to the amount expected had conventional crops been employed on the same area (Brookes and Barfoot 2018). In addition, relative to conventional crops, 2945 million kg of carbon dioxide have not been released into the atmosphere, because of the fuel saved from fewer runs needed to spray GM insect-resistant maize and cotton (Brookes and Barfoot 2018). Despite this, for a diversity of reasons, some still regard the technology with suspicion, thus giving cause for greater technological regulatory delays and more barriers to international trade, which usually result in forgone benefits (Smyth 2017b). To a certain extent societal concerns regarding the safety of food derived from GM crops is understandable, given the public’s limited knowledge (Popek and Halagarda 2017). It would be overly optimistic, to expect the general public to be able to differentiate between GM and genome-edited crops in the absence of transparent information or public education efforts. Furthermore, the politicization of risk has created a divergence of regulatory approaches: the major crop exporting nations (e.g. North America, Australia, Argentina, Brazil) use a pragmatic, science-based approach while importers (e.g. the EU and others) have been more cautious, using science tempered by political considerations (Smyth and Phillips 2014).

#### Genome editing

Mutagenetic technologies advanced rapidly in the 2000s into what is now known as genome editing, which refers to point-specific mutations in the genome, such as site-directed nucleases (SDN) and oligo-directed mutagenesis (ODM). The SDN technology includes a number of variants with analogous function: transcription activator-like effector nuclease (TALEN), zinc-finger nucleases (ZFN) and meganucleases, culminating in the discovery of CRISPR (Doudna and Charpentier 2014). SDNs allow for the introduction of small precision modifications (SDN 1 and 2) of larger pieces of DNA or introduction of complete genes at a predetermined location (SDN3). Genome editing has numerous advantages over earlier technologies, most significantly that it allows for targeted, single gene mutation across the entire plant genome. The CRISPR suite of breeding tools offers an easier, more versatile and accurate form of mutagenesis that facilitates transfer of the desired trait to progeny without losing any efficacy (Georges and Ray 2017). This technology is able to perform mutations to a specific site within the targeted gene, making the effects on the plants more significant (Song et al. 2016), as it can be programmed to target specific segments of genetic code or edit DNA with greater accuracy (Barrangou 2015). In addition to crop breeders, this is particularly attractive to animal and medical scientists as they anticipate the potential for treating disease through genome editing. Importantly, it holds great potential for public sector plant breeding in developing countries, allowing for local and regional solutions to improving food security. For example, a Chinese research group (Miao et al. 2018) has already made use of CRISPR/Cas9 technology to create a rice variety that yields 25–31% more output than conventional varieties. This could have profound implications for food security.

Nonetheless, for all the benefits CRISPR/Cas9 seems capable of providing, Smyth (2017a) identifies that not all governments will embrace this technology. One reason is that applications of genome editing yield different outcomes. Some modifications (SDN 1 and SDN 2) can be generated by chemical mutagenesis, radiation or natural mutations, with the resulting organisms similar to those obtained by traditional breeding or classical mutagenesis (e.g. glyphosate-resistant CRISPR rice for weed control). Other repair mechanisms involve delivering foreign DNA (SDN 3), with the outcome that the resulting products would be viewed as transgenic for regulatory risk assessments. In 2016, in response to a lawsuit launched by nine non-governmental organizations, a French court referred a request to regulate genome-edited varieties as GMOs to the Court of Justice of the European Union (CJEU) for an interpretation of European Law pertaining to new plant breeding techniques, especially CRISPR/Cas9. On 25 July 2018, the CJEU ruled that mutagenic crops are subject to the European Union’s regulatory system in the same way as transgenic GM organisms (CJEU 2018). The ruling refers to modern forms of mutagenesis, and it did not clarify any genome-editing exemption. Regrettably, additional clarity will not be forthcoming as in January 2019 the European Commission announced that no new legislation regarding the regulation of crop technologies is planned, resulting in the CJEU ruling being binding in its current form (Livingstone 2019).

## Method

An online survey was conducted between January and April 2018 to gather expert opinions on potential benefits of NBTs. The survey was emailed to a panel of 507 international experts (scientists, government officials, agribusiness professionals, etc.) with related backgrounds and experience in biotechnology. The survey instrument is part of a multi-year survey project investigating expert opinions regarding the application of NBTs and their potential to enhance global food security. The expert panel was obtained from a contact database that was constructed using emails of participants for a number of conferences on biotechnology organized by the researchers over the past 15 years, and of experts from online searches (i.e. websites of universities, research institutions, biotech companies and government agencies). This panel allowed us to reach a large number of international experts in the field of study.

Our study (BEH 97) was deemed exempt from full ethics review by the Behavioural Ethics Board at the University of Saskatchewan on April 7, 2015, on the basis that the participants, as experts, were not themselves the focus of the research.^1^ Nevertheless, our online survey presented participants with a standard consent statement describing the study, identifying the absence of known risks associated with participation, and a reminder that participation was voluntary and responses would be anonymous and confidential. Upon expression of consent, participants were presented with the survey.

The survey included three opinion-based questions. The first and second questions were phrased as short statements, soliciting responses on a five-point Likert scale. Respondents were invited to provide their agreement with a list of different potential benefits of genome-edited crops compared to GM and CONV alternatives, along with their confidence level on a five-point Likert scale (1 being least confident and 5 being most confident). Benefits of genome-edited crops were measured using 15 agronomic, environmental, and socio-economic factors. We acknowledge that these benefits can be achieved ‘theoretically’ by any of these three technologies, however, feasible outcomes of some techniques are restricted by regulatory burden, high costs, lengthy process and low consumer acceptance. The third question asked about the anticipated significance (i.e. minor, moderate or major) of a number of potential regulatory events in influencing where and how genome editing will be developed and used in agriculture.

At the end of the survey, a hypothetical binary choice question was asked to test for temporal preferences (using a lottery prize). Participants were asked how they would use an imaginary prize of $5000: for a summer vacation or for retirement saving. The question was used to compare the benefits-related questions between two groups of experts: those who tend to exhibit long- versus short-term preferences. This question was used only for classification purposes and it is not intended as a substitute for theoretical discounting models. It was useful in the context of our study to have a simple, univariate measure of discounting that was not tied to any specific theoretical framework.^2^

## Results and analysis

The survey was completed by 114 respondents, resulting in a response rate of 22.5%. The sample is dominated by male subjects (79%), aged between 45 and 65 years (70%). Fifty-three percent of participants reside in North America, 28% in Europe, and the remainder from the rest of the world (4% from Africa, 6% from Asia, 4% from Oceania and 5% from Central and South America). Sixty-three percent identified themselves as scientists and 37% as non-scientists (government officials, agribusiness delegates, etc.). Forty percent work for industry, 26% for university, and 20% for government.

As clarified earlier, not all genome-edited crops fall into the same category. Results of previous surveys within this project show that expert decision makers agree that some genome-edited crops are transgenic and thus should be regulated as GM while others should not (Lassoued et al. 2018). Having this result in mind, the analysis below compares site-specific genome-edited crops (free from foreign DNA) to GM and CONV counterparts. On average, the mean level of benefits of genome-edited crops compared to GM (CONV) alternatives was 3.6 (3.75) with a standard deviation (SD) of .73 (.73) on the one-to-five scale. This reveals that experts largely agree on the potential benefits of genome-edited crops in terms of agronomic performance (disease resistance, drought tolerance, high yields, etc.), final product quality (nutrition, shelf life, etc.), climate change resilience, and global food security (Table 1). More than half of the sample was undecided (neutral or cannot tell) about the impact of genome-edited crops on environmental sustainability (e.g. reduced agri-food waste, enhanced biodiversity) and economic advantages for farmers (i.e. higher returns). As for the impact of genome-edited crops on consumer confidence, environmental footprint and trade, a plurality tends to be neutral or cannot tell at 46%, 45% and 44%, respectively.

**Table 1:** Expert opinions of impact of genome editing compared to GM and CONV (N = 114)

Do you agree or disagree genome-edited crops will generate more benefits compared to	Strongly disagree/disagree		Neutral/can’t tell		Strongly agree/agree	
	GM	CONV	GM	CONV	GM	CONV
Improved resistance to diseases	11	4	27	15	62	81
Increased drought tolerance	11	6	31	17	58	77
Improved processing qualities	11	6	31	23	58	71
Longer shelf life and storability	11	5	33	20	56	75
Higher yields	10	6	36	18	54	76
Better nutritional or functional qualities	11	5	35	20	54	75
Improved climate change resilience	11	9	36	17	53	74
Increased food security	9	6	39	29	52	65
Lower production costs	12	9	40	34	48	57
Improved consumer confidence	16	34	38	40	46	26
Lower environmental footprint	14	8	41	24	45	68
Freer international trade	17	35	39	39	44	26
Higher farmer income	11	10	51	37	38	53
Reduced agri-food waste	15	10	50	41	35	49
Enhanced biodiversity	17	19	54	42	29	39

The scale options “Strongly agree” and “Agree” were grouped together to increase the cell count. Same for “Strongly disagree” and “Disagree”, and for “Neutral” and “Cannot tell”. The recoding does not alter the result interpretation

Similarly, the majority of experts believe that genome editing offers more opportunity than CONV to produce crops with improved agronomic performance, product quality, farmer profitability (lower production cost, higher income), climate resilience, and global food security. These advantages are derived from the accuracy and precision of genome-editing technology that some say could help save years of development time and lower the cost to produce certain traits in crops. Yet, opinions on socio-political and environmental questions are less consistent. While 68% of the participants agree/strongly agree that genome editing will enable breeders to lower agriculture’s environmental footprint, the sample is divided into those who are optimistic that genome editing could be more effective than CONV breeding in creating varieties that would reduce agri-food waste (49%) and enhance biodiversity (39%) and those who are neutral (41% and 42%). Experts are also divided about whether genome-edited crops will enhance consumer confidence or open foreign market access: 35% and 34% think it will not help at all and 40% and 34% are uncertain. Only about a quarter of the sample (26%) agree/strongly agree that genome-edited crops might help resolve trade restrictions and enhance consumer confidence. Genome editing is still a relatively new technology. Like any innovation, its effects are still somewhat speculative. Many panelists commented that while existing biotechnologies can deliver similar benefits to genome-edited crops, products of genome editing might gain better socio-political advantages.

Overall, experts converged on a consensus that genome editing would offer better agronomic performance and product quality than the alternatives. Yet this does not imply that genome-editing technology is the ideal substitute for GM and CONV breeding techniques—they can and probably will coexist. GM and CONV still deliver important benefits, but genome editing would appear to deliver certain benefits better and faster thanks to its precision (advanced knowledge in genomics) and the potential lower regulatory oversight. Innovative plant breeding does not necessarily mean abandoning earlier breeding methods as different technologies might perform better than others for different breeding targets. For instance, the GM approach, despite being less precise, has the potential to perform better than genome editing to control certain viruses (see e.g. Ali et al. 2016; Lassoued et al. 2018). Yet, GM technology has been facing several regulatory and social barriers that limited the agronomic (and subsequently socio-economic) potential in rice and wheat, the world’s most important staple grains.

Our results clearly show a divergence in expert opinion about the potential for genome-edited crops to contribute to more effective international trade or enhanced conservation, biodiversity or consumer trust. The debate surrounding the success of genome-edited crops is now not a matter of technology, but a matter of public acceptance and legal clarity (e.g. Wolt and Wolf 2018; Araki and Ishii 2015; Ishii and Araki 2016b). Related to this, one respondents commented: “Gene-edited products can provide almost any of the qualities listed above—but so can genetically modified crops. The only advantage to the gene-edited crops OVER genetically modified crops is (hopefully) less regulation and hence they should have freer international trade.” Another participant said: “Gene editing has a chance to progress faster than GM but only because of perception and political issues and not because it’s better.” Regulation and social acceptance of novel breeding technologies will be key to their development and to the commercialization of derived crops, including genome-edited crops (Lassoued et al. 2018).

### Potential regulatory response

Experts were asked about the potential impact of various scenarios in determining the adoption and use of NBTs in agriculture. As shown in Table 2, the majority of experts indicated that the regulations for health and safety, followed by export trade rules, consumer acceptance, and engagement of the media will all play major roles in determining where and how NBTs will be developed and used in agriculture. Experts are less convinced about how farmers and local markets will respond. These results suggest that the adoption of NBTs might not be constrained by availability of research funding and skills but will be influenced by socio-political factors (e.g. Wolt and Wolf 2018).

**Table 2:** Potential impact of various scenarios in determining the adoption and use of NBTs in agriculture (%)

How significant do you think the agents below will be in determining where and how NBTs will be developed and used in agriculture?	Minor role	Moderate role	Major role	Uncertain
National or regional regulations for health and safety	4	17	74	5
Export markets	10	29	56	5
End-users/consumers	15	26	53	6
The media	14	31	51	4
Plant breeders	20	36	40	4
Industry or product standards	12	41	40	7
Research funders	19	45	31	5
Wholesale trade rules	17	39	31	13
Food processors	20	46	30	4
Research managers or leaders	30	38	27	5
Host research institutions	24	43	26	7
Farmers	33	41	21	5
Local markets	38	41	15	6

### Group differences

Table 3 parses the survey responses on the relative benefits of genome-edited crops using three categorical (independent) variables: expertise (scientists: 63% vs. non-scientists: 37%), region of residence (NA: 53%, Europe: 28% and the ROW: 19%) and time preference (short-run: 49% vs. long-run: 51%). As the dependent variable—benefits—is continuous (calculated as the mean of the five-point Likert scale of agreement on the benefits of genome-edited crops taking into account all 15 items of Table 1), a *t* test was used to test for differences in the means for the two binary categorical variables (i.e. time preference and expertise) and an analysis of variance (ANOVA *F*-test) for the non-binary categorical variable (i.e. region of residence).

**Table 3:** Relative benefits of genome-edited crops by group type

Grp type	Compared to GM crops			Compared to CONV crops		
	N	M	SD	N	M	SD
*Time preference*						
Short term	54	3.58	.69	55	3.66	.80
Long term	54	3.56	.75	55	3.83	.64
*p* value (*t*-test)	.904			.220		
*Expertise*						
Scientist	71	3.50	.74	71	3.7	.74
Non-scientist	37	3.70	.67	39	3.79	.72
*p* value (*t*-test)	.187			.613		
*Region*						
NA	55	3.66	.75	57	3.83	.73
Europe	31	3.41	.75	31	3.56	.81
ROW	22	3.55	.60	22	3.78	.58
*p* value (*F*-test)	.301			.255		

The independent *t*-test indicates the respondents’ time preference (short or long-term) generated no significant difference in the anticipated mean benefits of genome-edited crops compared to GM crops (t_(106)_ = .121, *p* = .904) or to CONV crops (t_(108)_ = − 1.235, *p* = .220). In effect it can be concluded that time preference has no effect on the perception of the benefits of genome-edited crops, as both groups reported similar mean benefits regardless of their time preferences. The independent *t*-tests indicate that respondents’ expertise (scientist or non-scientist) caused no significant difference in the expected mean benefits of genome-edited crops compared to GM crops (t_(108)_ = − 1.327, *p* = .187) and to CONV crops (t_(108)_ = − .508, *p* = .613). Hence, expertise seems to have no effect on the perception of the benefits of genome-edited crops, as both groups of experts reported similar mean level of benefits. The *F*-test for the ANOVA for regional effects (NA, Europe and ROW) reveals no statistically significant difference in the mean benefits of genome-edited crops compared to GM crops (F_(2)_ = 1.214, *p* = .301) and to CONV crops (F_(2)_ = 1.383, *p* = .255) among different regions. These results suggest that where one lives has no effect on perceptions of the benefits of genome-edited crops, as the three groups reported similar anticipated mean benefits. In fact, the majority of experts are relatively optimistic about the potential of genome-edited crops, regardless of where they live, with the exception that diverging public acceptance could result in contradictory regulatory choices in different countries, which might create market barriers (e.g. Shao et al. 2018).

## Conclusion

This paper reports the opinions of biotechnology experts, both in the life and social sciences, on genome-edited crops and technologies. The primary finding of this research is that there is a consensus among experts on the expected greater agronomic performance and product quality of site-specific edited crops—those free from foreign DNA will be more competitive than GM and CONV counterparts. Such new crops have the potential to deliver a greater diversity of traits and varieties in a quicker and less costly way. Optimism is hampered only by the non-technical dimensions of the technologies. That is, an ensemble of accompanying socio-economic considerations will be key in consumer and regulatory perceptions of this technology and will define their ultimate utility. A majority of experts indicated that the regulations for health and safety, followed by export markets, consumers, and the media will play a major role in determining where and how NBTs will be developed and adopted in agriculture. Regardless of potential and already perceived genome-edited crop benefits, debate around consumer acceptance and trade will undoubtedly surface and determine acceptance or rejection. Public understanding of the difference between some genome-edited crops (i.e. non-transgenic) and GM crops is critical for the development of the technology. The good news is that recent research has shown that consumers of certain countries are more willing to consume food derived using CRIPSR as compared to GM food, which may indicate an opportunity to reduce the skepticism about agriculture biotechnology (Shew et al. 2018). Sustaining this support could be a challenge.

As the experts indicated, genome editing from a technological perspective is but another tool for plant breeders to use in the development of new varieties. Experts identified that the technology could be most valuable in speeding up regulatory approval. Numerous countries have indicated that if no foreign DNA is present in a crop variety, it will not require any additional regulatory oversight or risk assessment. Currently only the European Union (EU) has judged that even in the absence of foreign DNA any genome-edited variety must be regulated as equivalent to transgenic GMO varieties. The problem is that this diverging interpretation of the risks is more political than technological. Since the establishment of the European Food Safety Authority in 2003, the EU has integrated scientific risk assessment with political risk expediency, for the most part generating regulatory gridlock, with only a single GM crop variety approved in 15 years. Going further down this path will be problematic.

At the time the survey was conducted, the CJEU had not ruled on NBTs. Now that there is precedent of a regulatory setback for NBTs, it is possible that the opinion of experts and consumers has shifted. Scientists have become increasingly vocal about the long-term adverse impacts this ruling could have on crop variety research and development within the EU. Indeed, scientists representing over 80 scientific research centres and institutions within Europe signed a petition calling for the European Commission to use science-based regulations in the assessment of NBT crop varieties (VIB 2018). However, based on past experience when experts and consumers have disagreed, the concerns expressed by experts might not override consumer perspectives in any meaningful way. Further research will be needed to see how consumer acceptance of genome-edited crops evolves and whether experts have any say in how or where this technology might be used in support of better food outcomes.

## Electronic supplementary material

Below is the link to the electronic supplementary material.

Supplementary material 1 (PDF 107 kb)

## Footnotes

1 Per the Tri-Council Policy Statement: Ethical Conduct for Research Involving Humans, December 2014, Exemption Article 2.1.

2 We asked nine questions covering different topics (health, financial situations, etc.) to gauge temporal preferences in the survey. However, we present here the results using only one question as the results of the other questions yield extremely unbalanced sub-samples: the majority of experts exhibited long-term preferences.
