# Supplementary material for: Benefits of genome-edited crops: expert opinion
Source: Transgenic Res. 2019 Mar 4;28(2):247–56. doi: 10.1007/s11248-019-00118-5 (PMC6440930; doi:10.1007/s11248-019-00118-5)
Supplement: Supplementary file 1 — Supplementary material 1 (PDF 107 kb) [file 11248_2019_118_MOESM1_ESM.pdf]

Benefits of New Breeding Techniques Survey

Consent_Text

Dear participant,

We appreciate your participation in our seventh quarterly survey that includes questions related to **the benefits of New Breeding Techniques (NBTs)**, in addition to general questions about **time preferences**. The questionnaire is part of a three-year project on risk decision-making regarding NBTs. You have already completed at least one survey with us, and your responses have been invaluable in moving the project forward.

The multi-year survey project is investigating risk preferences among knowledgeable experts regarding innovative technology applications in the agri-food industry. The lead researchers for this project are: Dr. Stuart Smyth (stuart.smyth@usask.ca, (306) 966 2929) and Dr. Peter Phillips (peter.phillips@usask.ca, (306) 966 4021). They can be contacted should you have any questions or comments. Any questions regarding your rights as a participant may be addressed to the University of Saskatchewan Research Ethics Office ethics.office@usask.ca; (306) 966-2975. Out of town participants may call toll free (888) 966-2975.

As an expression of our gratitude, we will ensure you are granted access to all publications, reports and press releases prior to their publication.

This survey is hosted by Voxco, a Canadian-owned and managed company whose data is securely stored in Canada. Please consider printing this page for your records.

There are no known risks to participating in this survey; however, as with any online activity the risk of breach of confidentiality is always possible.

In order to complete this survey, you may be required to answer certain questions; however, you are never obligated to respond and you may withdraw from the survey at any time by closing your internet browser.

By selecting next and completing this questionnaire, your free and informed consent is implied and indicates that you understand and accept the above conditions of participating in this study.

DESCRIPTION

Background:

New Breeding Techniques (NBTs) including gene editing have emerged as the result of advances in scientific research. They enable more precise and faster changes in the plant’s

genome than conventional plant breeding techniques, which use chemical and radiation processes to alter the genetic characteristics of plants (EFSA, 2016)[1]. As such, they have a significant potential for the plant breeding and agri-food industries, as they entail technical advances, economic savings and the improvement of crop characteristics (Madre and Agostino, 2017)[2].

We are interested in the benefits of gene-edited crops compared to transgenic and conventional counterparts.

[1] http://www.europarl.europa.eu/RegData/etudes/BRIE/2016/582018/EPRS_BRI(2016)582018_EN.pdf
[2] http://www.farm-europe.eu/travaux/new-plant-breeding-techniques-what-are-we-talking-about/

Q1_1

Case 1: Do you agree or disagree that gene-edited products will generate MORE benefits (listed below) than **genetically-modified crops**?

Benefits	**Strongly disagree 1**	**Disagree 2**	**Neither agree nor disagree 3**	**Agree 4**	**Strongly Agree 5**	**Cannot tell**
Lower production costs						
Improved consumer confidence						
Higher farmers' income						
Lower agriculture's environmental footprint						
Enhanced biodiversity						
Improved climate change resilience						
Increased food security						
Freer international trade						
Improved resistance to diseases						
Increased drought tolerance						
Higher yields						
Better nutritional or functional qualities						
Longer shelf life and storability						
Improved processing qualities						

Reduced agrifood waste						
Other (Please specify ……)						
Other (Please specify ……)						
Other (Please specify ……)						

Q1_2

On a scale of 1 to 5, 5 being most confident, please indicate how confident you are with your responses above?

**(least confident) 1**	2	3	4	**(most confident) 5**

Q1_1Open

Comments (if any):

[textbox]

Q1_3

<u>Case 2:</u> Do you agree or disagree that gene-edited products will generate MORE benefits (listed below) than **conventional crops**?

Benefits	**Strongly disagree 1**	**Disagree 2**	**Neither agree nor disagree 3**	**Agree 4**	**Strongly Agree 5**	**Cannot tell**
Lower production costs						
Improved consumer confidence						
Higher farmers' income						
Lower agriculture's environmental footprint						
Enhanced biodiversity						
Improved climate change resilience						
Increased food security						
Freer international trade						
Improved resistance to diseases						
Increased drought tolerance						
Higher yields						
Better nutritional or functional qualities						

Longer shelf life and storability						
Improved processing qualities						
Reduced agrifood waste						
Other (Please specify ……)						
Other (Please specify ……)						
Other (Please specify ……)						

Q1_4

On a scale of 1 to 5, 5 being most confident, please indicate how confident you are with your responses above?

**(least confident) 1**	2	3	4	**(most confident) 5**

Q1_3OPEN

Comments (if any):

[textbox]

Q1_5

Did you base your previous answers on a particular crop or set of crops?

- No
- Yes. Please specify which one(s)? [textbox]

Q2

There is considerable debate about how NBTs, including gene editing, should be regulated. Regulation can occur at many levels. How significant do you think the agents below will be in determining where and how gene-editing will be developed and used in agriculture?

**Potential regulatory agents**	**Minor role**	**Moderate role**	**Major role**	**Uncertain**	**Why? Where?**
Research managers or leaders	o	o	o	o	[textbox]
Research funders	o	o	o	o	[textbox]
Host research institutions	o	o	o	o	[textbox]
National or regional regulations for health and safety	o	o	o	o	[textbox]
End-users/consumers	o	o	o	o	[textbox]
The media	o	o	o	o	[textbox]

Wholesale trade rules	o	o	o	o	[textbox]
Food processors	o	o	o	o	[textbox]
Plant breeders	o	o	o	o	[textbox]
Farmers	o	o	o	o	[textbox]
Industry or product standards	o	o	o	o	[textbox]
Local markets	o	o	o	o	[textbox]
Export markets	o	o	o	o	[textbox]
Other (Please specify ……)	o	o	o	o	[textbox]
Other (Please specify ……)	o	o	o	o	[textbox]
Other (Please specify ……)	o	o	o	o	[textbox]

Q3_Description

Using the sliders below, who do you think will benefit the most if gene-edited crops are commercialized?

Q3a

[0-100 slider; default value at 50]

Low-income countries --- High-income countries

Q3b

[0-100 slider; default value at 50]

Local farmers --- Multinational agribusinesses

Q3c

[0-100 slider; default value at 50]

Consumers --- Agrifood industry

Q4_DESCRIPTION

We would like to ask you some general questions about your temporal preferences. In the **hypothetical** situations below, you are invited to make a choice between two options.

Q4_1

Imagine you are rewarded at the end of this survey. Do you prefer?

- A US$50 gift card once you finish.
- A US$100 gift card a year from now, once the survey project closes.

Q4_2

If your phone gets broken, would you prefer to replace it with:

- A cheap, less durable phone.
- Spend more on longer lasting phone.

Q4_3

If you win the “lottery for life”, would you choose?

- US $25,000 a year for life (guaranteed for 20 years).
- US $500,000 Lump sum payment.

Q4_4

When you go for dinner at a restaurant, do you usually prefer?

- Having a calorie rich desert, such as chocolate cake.
- Turning down the desert for better health.

Q4_5

Would you like to have:

- 15 min massage now, or
- 20 minute massage in an hour?

Q4_6

Would you choose between:

- Not getting a flu shot with the likelihood of getting the flu and having to take 3 days sick leave later in the winter, or
- Getting a flu shot before the flu season and having one day of not feeling well later in the winter?

Q4_7

In your job, do you usually:

- Delay completing stressful tasks until the last minute, or
- Try to solve them as soon as possible?

Q4_8

Imagine you won a lottery prize of $5000. Would you use that for:

- Summer vacation, or
- Your retirement saving?

Q4_9

Do you have regular (medical) checkups?

- No
- Yes
